# Main Health-Promoting Compounds Response to Long-Term Freezer Storage and Different Thawing Methods in Frozen Broccoli Florets

**DOI:** 10.3390/foods8090375

**Published:** 2019-09-01

**Authors:** Huiying Miao, Jiayao Lin, Wei Zeng, Mengyu Wang, Leishuan Yao, Qiaomei Wang

**Affiliations:** 1 Key Laboratory of Horticultural Plant Growth, Development and Quality Improvement, Department of Horticulture, Zhejiang University, Hangzhou 310058, China; 2 Zhejiang Provincial Key Laboratory of Horticultural Plant Integrative Biology, Hangzhou 310058, China

**Keywords:** frozen broccoli, freezer storage, defrosting, glucosinolates, vitamin C, total phenols

## Abstract

The effects of long-term freezer storage and different defrosting methods on the retention of glucosinolates, vitamin C, and total phenols in frozen broccoli florets were investigated in the present study. Frozen broccoli florets were stored in a freezer at −20 °C for 165 days or subjected to defrosting by three different house-hold thawing methods (water, air, and refrigerator defrosting). Results showed that all glucosinolates were well preserved, while vitamin C and total phenols were reduced by less than 12% and 19% of the control, respectively, during the storage. Besides, refrigerator and air defrosting were better than water defrosting in glucosinolates retention, and refrigerator defrosting was the best in vitamin C preservation. No difference was observed in reserving phenolic compounds among the three methods. In conclusion, long-term freezer storage is an excellent way to preserve broccoli florets, and refrigerator defrosting is the best way to maintain the nutritional compounds in frozen broccoli florets.

## 1. Introduction

Horticultural crops, including fruits and vegetables, have been recognized for their human health benefits. They have high contents of non-nutritive, nutritive, and bioactive compounds. Bioactive compounds from horticultural crops have potent antioxidant, anticancer, and antimutagenic effects [1]. Broccoli (*Brassica oleracea* L. var. *italica*) is one of the most economically important vegetable crops and has been consumed worldwide by people from both western and eastern cultures due to its flavor as well as nutritional value [2]. As a member of the Brassicaceae, broccoli is rich in glucosinolates besides normal nutrient substances and bioactive molecules, such as proteins, minerals, vitamins, and phenolic compounds [3,4,5,6,7]. Glucosinolates are a group of sulfur-and nitrogen-containing secondary metabolites, and their hydrolysis products have been suggested to confer broccoli the function in lowering the risk of cardiovascular disease, cancer, and other chronic diseases [8,9].

However, broccoli has high respiration and transpiration rates, which makes it very perishable [10]. Lots of post-harvest handlings have been investigated to extend its shelf life [11]. Nevertheless, commercial freezing is one good method to maintain the quality of broccoli and to allow it to be available throughout the year [12]. During the commercial freezing process, broccoli always encounters pre-freezing and freezing handlings, which cause loss of quality. A great deal of research has been done to optimize pre-freezing and freezing approaches, including our previous study that proposed to employ steam blanching and ice-water cooling instead of the current blanching and cooling processing to minimize the reduction of glucosinolate contents [13,14,15,16,17,18,19].

After freezing, broccoli florets were either stored in a freezer or defrosted for consumption. During these processes, broccoli would suffer time and temperature abuses, and encounter physical as well as chemical changes. Rodrigues and Rosa (1999) have reported that short frozen storage was an excellent way to preserve glucosinolates in broccoli [20]. Concerning long-term freezer storage, studies mainly focused on sensory quality and antioxidants [16,17,21,22,23]. Till now, limited information is available about the retention of glucosinolates and other major health-promoting compounds in frozen broccoli florets upon freezer storage; the current study was, therefore, conducted to investigate the influence of long-term freezer storage on contents of glucosinolates, vitamin C, and total phenols in frozen broccoli florets. Besides, three house-hold defrosting methods (defrosting in water, air, and the refrigerator) were undertaken to make a comprehensive understanding of the effect of these handy and flexible defrosting methods on the retention of main phytochemicals in frozen broccoli florets.

## 2. Materials and Methods

### 2.1. Sample Collection and Preparation

#### 2.1.1. Freezer Storage 

Frozen broccoli florets (*Brassica oleracea* var. *italica* cv. Youxiu) with approximately 2 cm stalk were obtained from Haitong Food Group Co. in Cixi, Zhejiang province, China. The industrial pre-freezing processing and freezing handling conditions were described in our previous study [14]. Briefly, the florets were immersed in 1% NaCl for 15 min, washed, and then put into boiling water for 90 s. The hydro-cooling shower operating was used at 3 °C for 8 min to cool them down. At last, the florets were subjected to −26 °C for 8 min in an Individual Quick Freezing (IQF) fluidized tunnel system to obtain the frozen florets with a final temperature of −18 °C in the center of the florets. The frozen florets were stored at −20 °C in a freezer. Six florets were taken out 0, 10, 34, 70,102, 133, and 165 days after storage. Then, three of them were frozen in liquid nitrogen and stored at −80 °C for determining vitamin C, while the other three of them were freeze-dried (Vir Tis Inc., New York, NY, USA) for glucosinolate and total phenol content assays. 

#### 2.1.2. Defrosting Methods

Six frozen broccoli florets each were taken from −20 °C freezer on the day after their arrival, and defrosted using one of the following methods:

(1) Water defrosting: Samples were placed in 10 volumes of 18 °C water. After 5 min, the temperature of samples reached up to 10 °C, and defrosting was completed.
(2) Air defrosting: Samples were wrapped by polyethylene films and placed in a 20 °C chamber with 60% relative humidity. After 1.5 h, the temperature of samples reached up to 10 °C, and defrosting was completed.
(3) Refrigerator defrosting: The samples were wrapped by polyethylene films and placed in a 4 °C refrigerator. After 8 h, defrosting was stopped when the temperature of samples was higher than 4 °C.

The control samples were from frozen (not defrosted) samples. Half of the defrosted or frozen samples were then freeze-dried for glucosinolate and total phenol content assays, while the rest were frozen in liquid nitrogen and stored at −80 °C for vitamin C assay.

### 2.2. Determination of Glucosinolate Contents

Glucosinolate contents were determined according to the previous report with minor modifications [14]. In this study, samples were boiled in 5 mL water for 10 min, and then the supernatant was transferred to a new tube after centrifugation (5 min, 7000× *g*). The residues were boiled for another 10 min with water (5 mL). The combined supernatants were applied to a DEAE-Sephadex A-25 column, and the glucosinolates were purified and converted to desulphoglucosinolates, as described [14]. Samples were subjected to the high-performance liquid chromatography (HPLC) analysis by using an HPLC instrument (Shimadzu, Kyoto, Japan) with an SPD-M20A diode array detector. A hypersil C18 column (5 μm particle size, 4.6 mm × 250 mm; Elite Analytical Instruments Co. Ltd., Dalian, China) was used with a mobile phase of acetonitrile and water at a flow rate of 1 mL/min. The procedure employed isocratic elution with 1.5% acetonitrile for the first 5 min; a linear gradient to 20% acetonitrile over the next 15 min; followed by isocratic elution with 20% acetonitrile for the final 13 min. Absorbance was measured at 226 nm. The *Ortho*-nitrophenyl-β-d-galactopyranoside (Sigma, St. Louis, MO, USA) was used as an internal standard for HPLC analysis. Data were expressed as μmol/g dry weight (DW).

### 2.3. Determination of Vitamin C Contents

The concentration of vitamin C was evaluated according to a method described previously [24]. Samples were homogenized at 4 °C in 10 mL of 1% (0.01 g/mL) oxalic acid, and 5 mL of 1% oxalic acid were used to wash the residues twice. The combined extracts were then centrifuged at 7000 rpm for 10 min. The supernatant was filtered by a 0.45-μm cellulose acetate filter. The same system as the one used in the glucosinolate assay was employed to perform HPLC analysis, with a mobile phase of 0.1% oxalic acid at a flow rate of 1 mL/min. Absorbance values were collected at 243 nm. Standard (L-ascorbate; Sigma, St Louis, MO, USA) was prepared and used to identify and quantify the vitamin C. The results were expressed as mg/100 g fresh weight (FW).

### 2.4. Determination of Total Phenol Contents 

Folin–Ciocalteu reagent method was employed to measure total phenol content, and the absorbance at 765 nm was recorded [25]. In detail, samples were ground twice with 12.5 mL of 30% ethanol, centrifuged at 7000 rpm at room temperature for 10 min after they were incubated at 37 °C for 1 h, and then the supernatant was collected. Gallic acid was chosen as a standard, and the results were expressed as mg gallic acid equivalent (GAE)/g dry weight.

### 2.5. Statistical Analysis

The SPSS package program version 11.5 (SPSS Inc., Chicago, IL, USA) was selected to perform the statistical analysis. Differences were tested by using one-way analysis of variance (ANOVA), followed by the least significant difference (LSD) test at a 95% confidence level (*p* < 0.05). The values are reported as means of three replications with standard error for all results.

## 3. Results

### 3.1. Effects of Long-Term Freezing Storage on Main Health-Promoting Compounds in Frozen Broccoli Florets

#### 3.1.1. Glucosinolates

**Figure 1:**
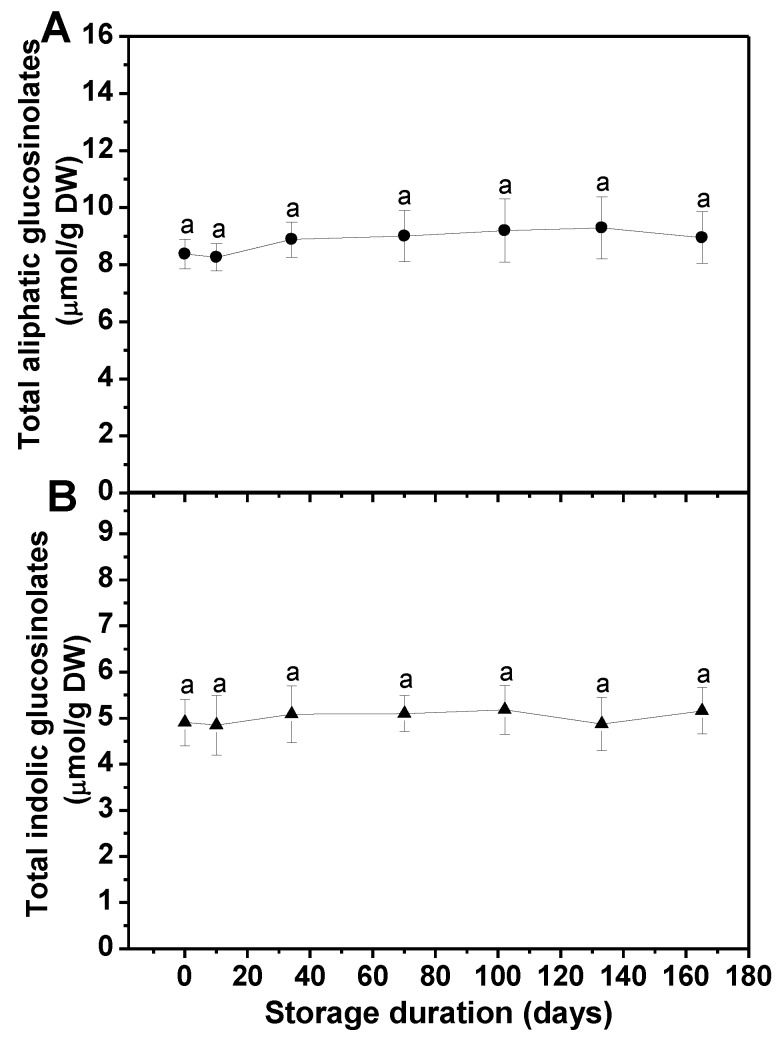
Effect of long-term freezer storage on the contents of total aliphatic (**A**) and indolic glucosinolates (**B**) in frozen broccoli florets. Each value is the mean ± standard error of three replicates (*n* = 3). Values not sharing a common letter are significantly different at *p* < 0.05. DW: dry weight.

The content and composition of glucosinolates were measured in frozen broccoli florets during long-term freezer storage. Being consistent with previous reports [14], nine glucosinolate profiles were identified. The predominant aliphatic glucosinolate was glucoraphanin, while the major indolic glucosinolate was glucobrassicin. On the whole, long-term freezer storage had no significant effect on the contents of total aliphatic and indolic glucosinolates (Figure 1). In detail, the amounts of all kinds of individual aliphatic glucosinolates remained constant during the 133-day storage, except for glucoiberin, whose content showed a significant increase of 40% over 34 days of storage (Table 1). At the end of the storage, the contents of progoitrin and glucoraphanin were markedly higher and lower than the initial, respectively. Even though, glucoraphanin was still the predominant glucosinolate profile by taking up 48% of the total aliphatic glucosinolates. The contents of four kinds of indolic glucosinolates that we identified remained unchanged during freezer storage, except that the level of 4-hydroxy glucobrassicin presented a considerable rise (33%) after 10 days of storage (Table 1). 

#### 3.1.2. Vitamin C and Total Phenols

**Figure 2:**
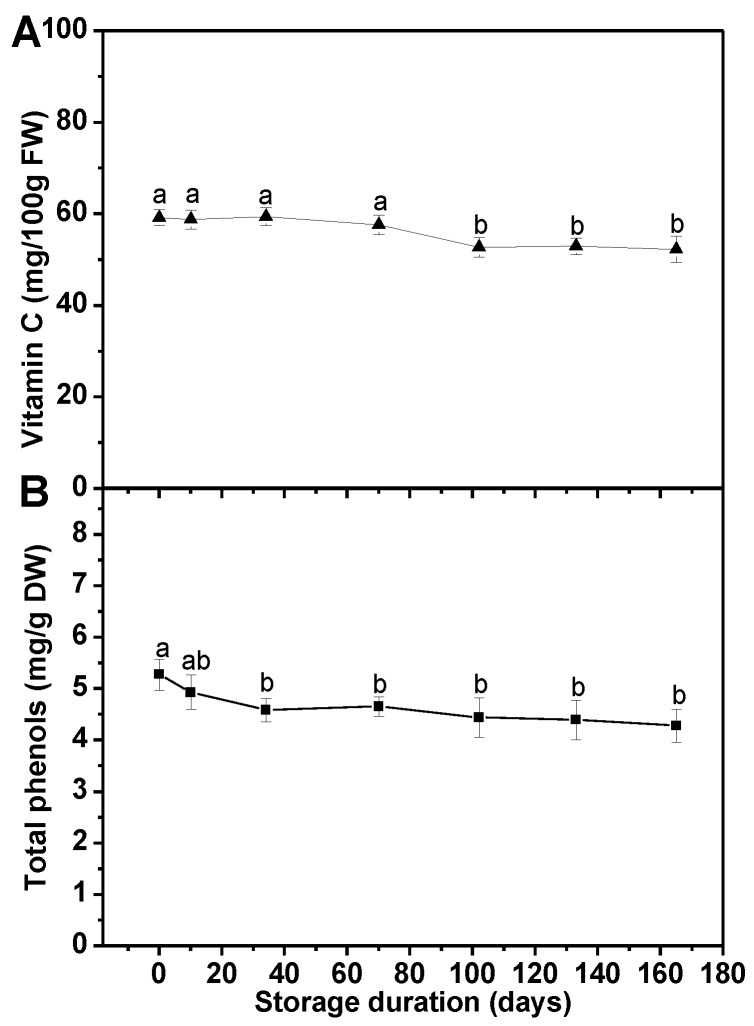
Effect of long-term freezer storage on the contents of vitamin C (**A**) and total phenols (**B**) in frozen broccoli florets. Each value is the mean ± standard error of three replicates (*n* = 3). Values not sharing a common letter are significantly different at *p* < 0.05. FW: fresh weight.

As shown in Figure 2, both vitamin C and total phenol compounds were stable during the first 10-day storage. No remarkable change was observed in vitamin C content after 70-day storage. As time went on, a slight decrease (11%) of vitamin C level in frozen broccoli florets happened after 102 days of storage, but the total loss of vitamin C was below 12% at the end of the storage. The amount of total phenols encountered a loss of 13% over 34 days of storage, and henceforth remained almost unchanged. After the long-term freezer storage, the total loss of phenolic compounds was less than 19% of the initial in frozen broccoli florets.

### 3.2. Effects of Different Defrosting Methods on Main Health-Promoting Compounds in Frozen Broccoli Florets

#### 3.2.1. Glucosinolates

**Figure 3:**
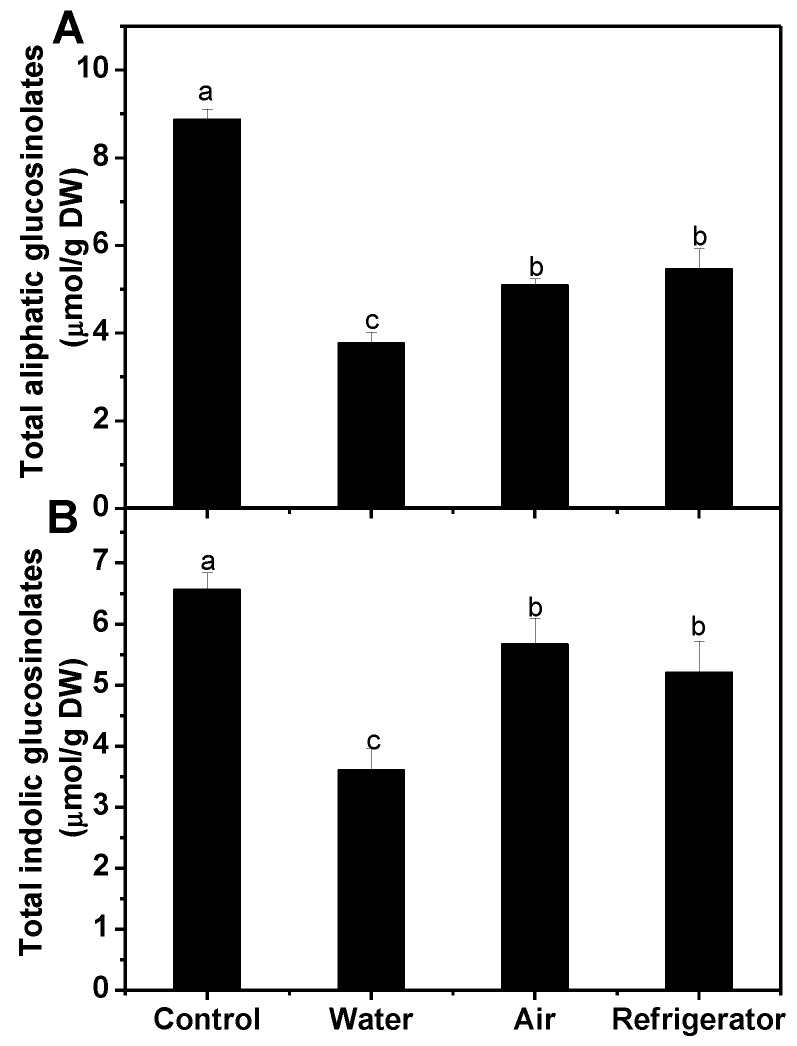
Effect of different defrosting methods on the contents of total aliphatic (**A**) and indolic glucosinolates (**B**) in frozen broccoli florets. Each value is the mean ± standard error of three replicates (*n* = 3). Values not sharing a common letter are significantly different at *p* < 0.05. DW: dry weight.

As showed in Figure 3, the content of total aliphatic glucosinolates dramatically dropped under three different defrosting treatments. Water defrosting resulted in a loss of 58% in total aliphatic glucosinolate content, while refrigerator defrosting and air defrosting led to a lower loss (38% and 42%, respectively). The loss of total indolic glucosinolates was not as severe as that of total aliphatic glucosinolates in response to all three defrosting methods. The amount of total indolic glucosinolates was markedly reduced by 45%, 14%, and 21%, respectively, upon the water, air, and refrigerator defrosting treatment. 

The levels of individual glucosinolates presented analogous changes with the total aliphatic and indolic glucosinolates upon three defrosting methods (Table 2). However, there was no noteworthy difference in the retentions of sinigrin, gluconapin, and 4-methoxy glucobrassicin among the three defrosting treatments. For glucobrassicin, a significant loss of 45% was found during water defrosting treatment, while its content was not remarkably affected by air and refrigerator defrosting.

#### 3.2.2. Vitamin C and Total Phenols

**Figure 4:**
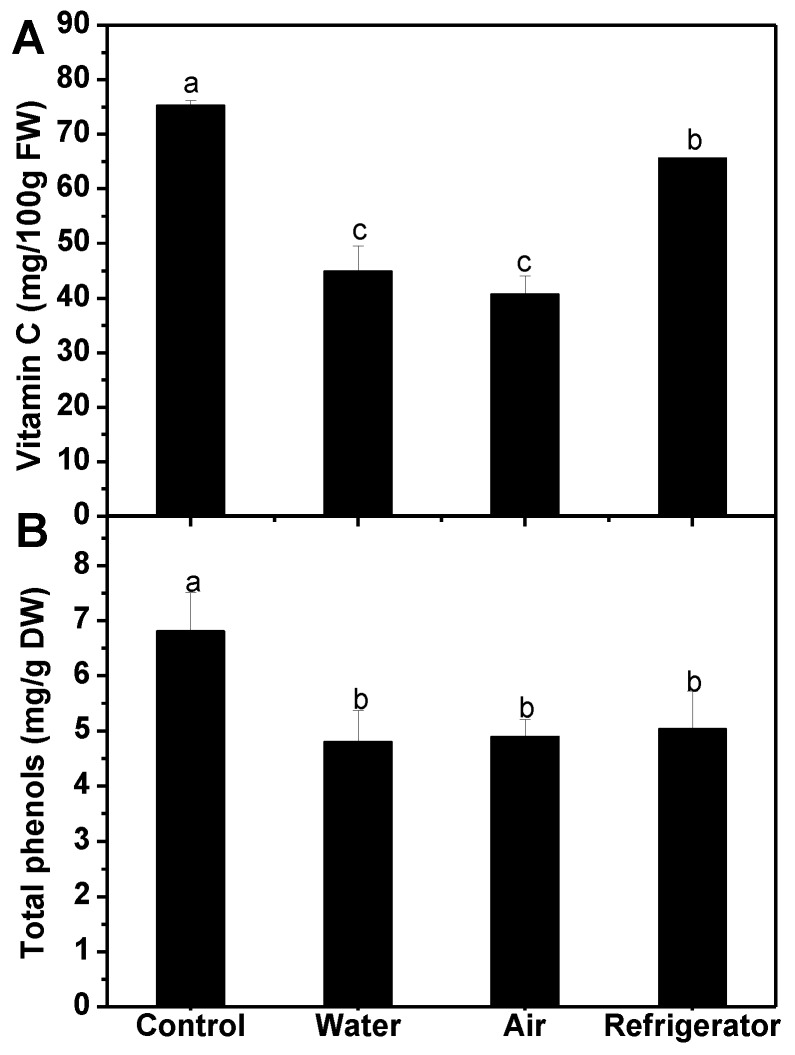
Effect of different defrosting methods on the contents of vitamin C (**A**) and total phenols (**B**) in frozen broccoli florets. Each value is the mean ± standard error of three replicates (*n* = 3). Values not sharing a common letter are significantly different at *p* < 0.05. DW: dry weight.

As shown in Figure 4, all defrosting treatments caused significant loss of vitamin C and total phenols. The highest retention of vitamin C was detected in broccoli florets under refrigerator defrosting (87%), followed by water defrosting and air defrosting (60% and 54%, respectively) (Figure 4A). However, no substantial difference was observed in the retention of phenolic compounds among the three different methods (Figure 4B). 

## 4. Discussion

The effects of long-term freezer storage and different defrosting methods on the retention of main health-promoting compounds, including glucosinolates, vitamin C, and total phenols in frozen broccoli florets, were analyzed in the current study. Our results indicated that the total aliphatic and indolic glucosinolate contents were not significantly affected by long-term freezer storage on the whole, which was consistent with the findings by Volden et al. (2009) in cauliflower (Figure 1) [26]. No dramatic change was detected in the levels of all kinds of individual aliphatic glucosinolates during the 133-day storage, while glucoiberin content showed a significant increase of 40% after 34 days of storage (Table 1). Interestingly, no significant changes were observed in the contents of sinigrin and gluconapin along with the whole storage, indicating that they were much more stable in response to long-term low-temperature storage. However, the predominant profile glucoraphanin suffered a decrease during the last month of storage. Regarding that the glucoraphanin and its degradation product sulforaphane are the major factors responsible for the anticancer property of broccoli [27], the freezer storage period is better not to be longer than 4.5 months. For individual indolic glucosinolates, their contents were not severely changed generally, except for 4-hydroxy glucobrassicin, which showed an increase over 10 days of storage. However, this would not result in a change in the total indolic glucosinolate level since its relatively low content. All in all, our findings suggest that long-term freezer storage is a good way to reserve the glucosinolates in frozen broccoli florets. 

The amounts of vitamin C and phenolic compounds in frozen broccoli florets upon long-term freezer storage were analyzed in this study as they are crucial antioxidants [28]. Water-soluble vitamin C is widely considered as an important marker to monitor the quality changes in the frozen chain. Irreversible oxidation mechanisms affect its concentration change, while temperature abuses are vital enhancer [29]. Former research in broccoli [23] and other vegetables and fruits [17,26,30,31] has put forward that frozen storage resulted in a decrease in vitamin C content. In this study, no remarkable change was observed in vitamin C content after 70-day storage, while a decrease of 11% occurred after 102 days of storage (Figure 2A). This loss was likely due to the oxidation reactions that could occur even at low temperatures [23]. Nevertheless, the total loss of vitamin C was less than 12% of the initial at the end of the storage. Previous studies on green bean, strawberry, and cauliflower have brought forward that the content of phenols was stable under long-term freezer storage [26,30]. However, a loss of total phenols was observed by us in broccoli florets after freezer storage for one month (Figure 2B). It has been noticed that the content of polyphenols in unblanched frozen strawberries underwent decreases during the storage at −20 °C for 6 months [32]. Therefore, the loss of phenols in the current study was probably due to limited enzymatic degradation, which was not completely inactivated during blanching. 

Defrosting is the reverse process of freezing, turning the ice crystals formed by freezing to soluble water again, which always brings about texture damage and drip loss [17,33]. In this study, the effect of three house-hold defrosting methods on glucosinolate retention in frozen broccoli florets was compared. Results showed that contents of total aliphatic and indolic glucosinolates were dramatically reduced by all defrosting methods, and the loss of total aliphatic glucosinolates was severer than that of total indolic glucosinolates (Figure 3). This was consistent with the findings by Vallejo et al. (2002) and Yuan et al. (2009) that indolic glucosinolates were more stable than aliphatic glucosinolates in response to some cooking methods [19,34]. Among the three defrosting methods, total aliphatic and indolic glucosinolates were better preserved when samples were defrosted in refrigerator and air rather than in water (Figure 3). As the degradation enzymes of glucosinolates have been inactivated during blanching [14,35], when frozen broccoli florets were immersed in water, quick defrosting caused severe cell disruption, and the subsequent water-based leaching might attribute to the severe loss of glucosinolates in water-defrosted samples [36,37]. For individual glucosinolates, water defrosting caused the greatest loss, while refrigerator defrosting was equal to air defrosting or even better in maintaining all kinds of individual glucosinolates, except for glucobrassicin and 4-hydroxy glucobrassicin (Table 2). It is known that the final retention of glucosinolates in defrosted broccoli florets is a result of many factors, including defrosting time, temperature, and the characteristics of different glucosinolates. More work is needed to explain why glucobrassicin and 4-hydroxy glucobrassicin are different from others in response to these house-holding defrosting methods. Nevertheless, it is highly recommended to defrost frozen broccoli florets in refrigerator or air to preserve more glucosinolates.

In the current study, refrigerator defrosting resulted in better retention of vitamin C when compared to air and water defrosting (Figure 4A). The work of Vallejo et al. (2002) in broccoli found that dipping in boiling water for 5 min led to a loss of 8.3% of total vitamin C into water [34]. Villarreal-Garcia et al. (2015) introduced the frozen broccoli florets in the boiling water for 40 s, and about 10% of vitamin C was lost [37]. Moreover, even washing treatment could cause considerable loss of vitamin C in broccoli florets [14]. All of the above suggested that water-soluble vitamin C was easier to lose when water-based handling was adopted, which might explain why water defrosting resulted in fewest vitamin C retention in the current study. Besides, higher vitamin C concentration was detected in samples defrosted in the refrigerator rather than in air, which was similar to the observation of Reis et al. (2015), as the cool and light-free condition was beneficial to protect vitamin C from damage by oxidation [38]. For phenolic compounds, all defrosting treatments caused significant loss of them, and no remarkable difference was observed between refrigerator defrosting and air defrosting (Figure 4B). This was inconsistent with the results reported by Reis et al. (2015), who showed that refrigerated temperature could retain more total phenolics than room temperature [38]. This difference might be due to the different broccoli materials and longer refrigerator defrosting time employed in the current study. Although it has been concluded that microwave thawing was better than thawing at room temperature in the retention of total phenolics in strawberry [32], a little better-equipped kitchen would be needed. 

On the whole, the contents of glucosinolates and vitamin C were not changed significantly after 70-day freezer storage, and the decrease in all determined phytochemicals was slight at the end of 165-day storage, indicating that long-term freezer storage is an excellent way to preserve the nutritional value of broccoli florets. Also, refrigerator defrosting is comparable with air defrosting in glucosinolate as well as phenolic compound retention, while it is the best in vitamin C preservation. Therefore, it is recommended to leave the frozen broccoli florets in the refrigerator beforehand to get them defrosted.

## Figures and Tables

**Table 1:** Effect of long-term freezer storage on the contents of individual glucosinolates in frozen broccoli florets.

Duration of Storage (days)	Aliphatic Glucosinolates (μmol/g DW)					Indolic Glucosinolates (μmol/g DW)			
	GIB	PRO	SIN	GRA	GNA	4–OH GBS	GBS	4–OM GBS	NGBS
0	0.57 ± 0.05 b	2.29 ± 0.19 b	0.46 ± 0.04 a	4.89 ± 0.33 a	0.18 ± 0.03 a	0.24 ± 0.02 b	2.57 ± 0.17 a	0.43 ± 0.01 a	1.68 ± 0.23 a
10	0.67 ± 0.06 ab	2.18 ± 0.30 b	0.48 ± 0.08 a	4.76 ± 0.37 ab	0.18 ± 0.03 a	0.32 ± 0.01 a	2.62 ± 0.25 a	0.43 ± 0.02 a	1.55 ± 0.20 a
34	0.80 ± 0.08 a	2.78 ± 0.33 ab	0.51 ± 0.10 a	4.62 ± 0.31 ab	0.18 ± 0.05 a	0.32 ± 0.02 a	2.64 ± 0.22 a	0.44 ± 0.03 a	1.76 ± 0.22 a
70	0.77 ± 0.06 a	2.83 ± 0.36 ab	0.51 ± 0.03 a	4.70 ± 0.51 ab	0.21 ± 0.01 a	0.31 ± 0.02 a	2.63 ± 0.15 a	0.45 ± 0.05 a	1.78 ± 0.21 a
102	0.75 ± 0.09 a	3.05 ± 0.51 ab	0.53 ± 0.08 a	4.69 ± 0.42 ab	0.18 ± 0.04 a	0.31 ± 0.03 a	2.66 ± 0.26 a	0.46 ± 0.04 a	1.82 ± 0.13 a
133	0.78 ± 0.03 a	3.09 ± 0.49 ab	0.50 ± 0.08 a	4.74 ± 0.52 ab	0.18 ± 0.05 a	0.32 ± 0.01 a	2.54 ± 0.17 a	0.45 ± 0.02 a	1.65 ± 0.16 a
165	0.80 ± 0.05 a	3.15 ± 0.47 a	0.51 ± 0.13 a	4.31 ± 0.14 b	0.19 ± 0.03 a	0.30 ± 0.02 a	2.54 ± 0.20 a	0.44 ± 0.04 a	1.94 ± 0.19 a

Each value is the mean ± standard error of three replicates (*n* = 3). Different letters denote statistically significant differences among the different days at *p* < 0.05. GIB: glucoiberin; PRO: progoitrin; SIN: sinigrin; GRA: glucoraphanin; GNA: gluconapin; 4–OH GBS: 4-hydroxy glucobrassicin; GBS: glucobrassicin; 4–OM GBS: 4-methoxy glucobrassicin; NGBS: neoglucobrassicin; DW: dry weight.

**Table 2:** Effect of different defrosting methods on the contents of individual glucosinolates in frozen broccoli florets.

Defrosting Methods	Aliphatic Glucosinolates (μmol/g DW)					Indolic Glucosinolates (μmol/g DW)			
	GIB	PRO	SIN	GRA	GNA	4–OH GBS	GBS	4–OM GBS	NGBS
Control	0.80 ± 0.02 a	3.16 ± 0.10 a	0.49 ± 0.02 a	4.21 ± 0.09 a	0.21 ± 0.01 a	0.33 ± 0.02 a	2.94 ± 0.08 a	0.56 ± 0.03 a	2.74 ± 0.14 a
Water	0.29 ± 0.02 c	1.20 ± 0.08 c	0.24 ± 0.02 b	1.92 ± 0.13 c	0.11 ± 0.02 b	0.11 ± 0.01 d	1.64 ± 0.16 c	0.35 ± 0.07 b	1.50 ± 0.11 b
Air	0.33 ± 0.05 c	1.73 ± 0.09 b	0.27 ± 0.01 b	2.63 ± 0.08 b	0.14 ± 0.02 b	0.18 ± 0.01 b	2.23 ± 0.11 b	0.44 ± 0.05 b	2.80 ± 0.16 a
Refrigerator	0.48 ± 0.05 b	1.68 ± 0.18 b	0.28 ± 0.04 b	2.88 ± 0.21 b	0.14 ± 0.02 b	0.14 ± 0.01 c	1.92 ± 0.17 c	0.38 ± 0.06 b	2.56 ± 0.17 a

Each value is the mean ± standard error of three replicates (*n* = 3). Different letters denote statistically significant differences among the different defrosting methods at *p* < 0.05.
